# Infant Male Circumcision: Healthcare Provider Knowledge and Associated Factors

**DOI:** 10.1371/journal.pone.0115891

**Published:** 2015-01-30

**Authors:** Erin J. Starzyk, Michele A. Kelley, Rachel N. Caskey, Alan Schwartz, Joan F. Kennelly, Robert C. Bailey

**Affiliations:** 1 University of Illinois at Chicago, School of Public Health, Division of Community Health Sciences, Chicago, Illinois, United States of America; 2 University of Illinois at Chicago, College of Medicine, Department of Internal Medicine and Pediatrics, Chicago, Illinois, United States of America; 3 University of Illinois at Chicago, College of Medicine, Department of Medical Education, Chicago, Illinois, United States of America; 4 University of Illinois at Chicago, School of Public Health, Division of Epidemiology and Biostatistics, Chicago, Illinois, United States of America; Genentech Inc., UNITED STATES

## Abstract

**Background and Objectives:**

The emerging science demonstrates various health benefits associated with infant male circumcision and adult male circumcision; yet rates are declining in the United States. The American Academy of Pediatrics and the Centers for Disease Control and Prevention recommend that healthcare providers present evidence-based risk and benefit information for infant male circumcision to parent(s) and guardian(s). The purpose of this study was to assess providers’ level of infant male circumcision knowledge and to identify the associated characteristics.

**Methods:**

An online survey was administered to healthcare providers in the family medicine, obstetrics, and pediatrics medical specialties at an urban academic health center. To assess infant male circumcision knowledge, a 17 point summary score was constructed to identify level of provider knowledge within the survey.

**Results:**

Ninety-two providers completed the survey. Providers scored high for the following knowledge items: adverse event rates, protects against phimosis and urinary tract infections, and does not prevent hypospadias. Providers scored lower for items related to more recent research: protection against cervical cancer, genital ulcer disease, bacterial vaginosis, and reduction in HIV acquisition. Two models were constructed looking at (1) overall knowledge about male circumcision, and (2) knowledge about male circumcision reduction in HIV acquisition. Pediatricians demonstrated greater overall infant male circumcision knowledge, while obstetricians exhibited significantly greater knowledge for the HIV acquisition item.

**Conclusion:**

Providers’ knowledge levels regarding the risks and benefits of infant male circumcision are highly variable, indicating the need for system-based educational interventions.

## Introduction

Infant male circumcision (IMC) is generally enveloped in a complex web of cultural and religious beliefs and practices.[1] Whether to circumcise an infant is a multifactorial decision, and influenced by numerous factors, including but not limited to parents’ race, ethnicity, insurance status, socioeconomic status, hospital type, geographic region, and healthcare provider (HCP) practices.[2–4]

Existing evidence demonstrates that male circumcision (MC) offers numerous health benefits and protections against certain medical conditions including human immunodeficiency virus (HIV),[5–7] various sexually transmitted infections (STIs),[8–13] urinary tract infections (UTIs),[14] penile,[15, 16] cervical,[13] and prostate cancers,[17] and other penile dermatoses, as well as providing increased penile hygiene.[18, 19] Despite the emerging science demonstrating circumcision’s health benefits, IMC rates are steadily declining in the United States.[20–23]

The research examining IMC reveals that HCPs do not always discuss this procedure with expectant parent(s) or guardian(s). A 2012 study of parents found that 49% of the sample had not discussed the advantages and disadvantages of circumcision with a HCP. Moreover, discussing IMC’s benefits significantly influenced the parents’ decision to circumcise their son.[4] Researchers in Miami asked Hispanic providers about their IMC practices, and found that this procedure is not readily discussed nor recommended with their patients in the predominantly Hispanic community. This lack of discussion and recommendation may stem from providers’ low level of knowledge regarding IMC as well as the belief that patients are not interested in the procedure.[24] Another recent study in 2008, found that 22% of physicians, which included pediatricians, family practitioners, obstetricians/gynecologists, and internists did not feel qualified to discuss IMC with parents due to a lack of understanding of the risk and benefits associated with this procedure.[25] In 2010, the American Academy of Pediatrics (AAP) reported that 57% of pediatricians believed that the medical benefits of IMC are inconclusive; 30% believed that the benefits outweighed the risks associated with this procedure; and pediatricians discussed IMC significantly less in 2006 (66%) compared to 1997 (74%).[26] Current research indicates significant variation among HCPs’ communicative practices with parents regarding IMC, which may influence their decision-making process and stem from a lack of knowledge.

In 2010, the Centers for Disease Control and Prevention (CDC) emphasized the importance of providing evidence-based risk and benefit information for IMC and adult MC to HCPs and parents in order to facilitate informed decision-making and promote parent-provider communication.[27] In August 2012, the AAP published an updated IMC statement explaining that the benefits associated with this procedure outweigh the risks, specifically highlighting the protection against acquisition of HIV and specific STIs.[28] The AAP stated that providers have a responsibility to present unbiased and accurate information in an effort to aid parents with the IMC decision. The statement further explained that the current evidence provides justification that all parents should have access to this procedure and furthermore, a third party payer should cover the procedure’s costs.[28] The American College of Obstetricians and Gynecologists (ACOG) also endorses AAP’s new IMC statement.[29]

At this time, there are no universal communicative guidelines for this procedure as seen with other preference-sensitive decisions. Therefore, the communicative practices are left to the discretion of the individual HCP when disseminating anticipatory guidance related to IMC. Research indicates that variation in provider communication practices may influence parental decisions around IMC.[4] Further, past evidence indicates that HCP knowledge is critical in promoting effective communication and decision support.[30]

In view of the attention that IMC continues to garner coupled with the results of recent research showing the health benefits of MC and IMC, this is a particularly opportune time to explore providers’ IMC knowledge and identify characteristics associated with their knowledge levels. Therefore, we hypothesized that knowledge is an important contributor to promoting informed decision-making for IMC.

## Materials and Methods

### Ethics Statement

This study received ethical approval by the University of Illinois at Chicago’s Institutional Review Board (protocol 2011–0212), and all participants provided informed consent.

### Study’s Objective, Design, and Sample

The objective of this study was to assess HCPs’ level of IMC knowledge and identify the associated characteristics. The study population consisted of primary healthcare providers who may be involved with the IMC decision process and who treat parent(s) and/or guardian(s) during the prenatal care, delivery, and post-natal stages. The sample was recruited from the universe of eligible providers from the family medicine, obstetrics, and pediatrics departments within the studied urban academic medical center in Chicago, Illinois. Providers included faculty physicians, fellows, residents, and mid-level providers (nurse practitioners, physician assistants, and nurse midwives). At the time of the study, first year residents were two months into residency training and were not included in the participant pool due to the limited experience with the subject matter.

Initially, the first author compiled the list of prospective participants from the academic health center’s online databases, and then worked with a key stakeholder from each medical department to determine the final sampling frame. In an effort to maximize an optimal response rate, each of the key stakeholders sent an email explaining the purpose of the study and introducing this study’s first author to potential participants. Subsequently, the first author sent each potential participant an email asking for his or her participation in the study with a link to the online survey. Throughout recruitment, prospective participants received encouragement and reminder emails in an effort to maximize enrollment. The study offered a five dollar electronic gift card to either Starbucks or Amazon as a small incentive if the participant completed the survey.

### Instrument, Data Collection, and Statistical Analysis

This survey was administered using the SurveyGizmo online platform from September 2011 to November 2011. All data were imported into SAS software version 9.2 (SAS Institute Inc., Cary, NC) for analysis.

We determined that a sample size of 90 participants would be sufficient to provide 80% power to detect a moderate effect size of 0.30[31] using a multiple regression approach with a two-tailed test with nQuery software.[32] We expected a 55% response rate based on current evidence-based health services literature and past studies with HCPs.[33–40]

To measure the providers’ level of knowledge, a summary score was constructed from evidence-based literature in conjunction with MC experts.[3, 41] The knowledge score consisted of 17 items on the online survey. Respondents received one point for each correct answer, and zero points for responses that were incorrect or “don’t know”. Items included questions about health conditions associated with IMC and MC, religious and ethnic groups’ circumcision practices, and policy issues.

For the analysis, two models were constructed looking at (1) overall knowledge about IMC, and (2) knowledge about HIV acquisition. The HIV item asked participants the following:

Research in Sub-Saharan Africa suggests that male circumcision reduces the transmission of HIV by approximately:

□ 10%
□ 30%
□ 60%
□ 90%
□ Unsure/Don’t Know

We looked specifically at HIV knowledge separately because three recently published randomized controlled trials demonstrated that adult MC significantly protects heterosexual men against HIV acquisition by approximately 60% in SSA.^4–6^ These findings have received a great deal of attention in the scientific literature and among medical societies. Due to the importance of the findings and their salience, we examined the factors associated with the HIV knowledge item.

In order to identify what characteristics influence IMC knowledge, the study explored a variety of factors including: demographics (listed in Table 1) as well as medical experiences and circumcision practices, which included

1. If the HCP ever performed IMC.
2. If the HCP received formal IMC training.
3. If the HCP ever refused to perform an IMC.
4. If the HCP had a son today, would he choose to circumcise him.

Further, participants’ circumcision beliefs and opinions statements were examined and measured utilizing 5-point Likert items from ‘Strongly Disagree’ to ‘Strongly Agree’ as follows in Table 2. For the analysis, the belief variables were collapsed from five categories—(1) Strongly Disagree, (2) Disagree, (3) Neither Agree nor Disagree, (4) Agree, (5) Strongly Agree—to two categories: (1) Strongly Disagree, Disagree, Neither Agree nor Disagree (2) Strongly Agree, Agree.

**Table 1:** Participant Sample Demographics.

Characteristic	n	%
Gender		
Female	72	78
Male	20	22
Age (years)		
20–29	15	16
30–39	38	41
40–49	13	14
50–59	17	19
60+	6	7
Prefer not to answer	1	1
Missing	2	2
Race		
White/ Caucasian	48	52
Asian	19	21
Black/African American	9	10
Native Hawaiian	2	2
Other	9	10
Prefer Not to Answer	5	5
Ethnicity		
Not Hispanic or Latino	77	84
Hispanic or Latino	12	13
Prefer Not to Answer	3	3
Religious Affiliation		
Christian	46	50
Hindu	8	9
No religious affiliation	8	9
Jewish	7	8
Muslim	6	7
Agnostic	4	4
Buddhist	2	2
Atheist	2	2
Other	3	3
Prefer Not to Answer	4	4
Missing	2	2
Birth Country		
United States	66	72
Other	22	24
Missing	4	4
Marital Status		
Married	53	58
Single	24	26
Steady Partner	6	7
Divorced	4	4
Prefer Not to Answer	3	3
Missing	2	2
Children		
Yes	45	49
No	43	47
Prefer Not to Answer	2	2
Missing	2	2
Decade Completed Medical Training		
1970–1979	3	3
1980–1989	16	18
1990–1999	15	16
2000–2010	26	28
2010 or later	31	34
Missing	1	1
Position		
Faculty Physician	37	40
Resident	36	39
Fellow	3	3
Nurse Practitioner	5	6
Nurse Midwife	11	12
Medical Specialty		
Obstetrics	37	40
Pediatrics	28	31
Family Medicine	27	29
Years practicing at the studied institution		
0–1	11	12
2–5	41	45
6–10	17	18
11–20	12	13
20+	11	12
Currently also practice at another institution		
No	78	85
Yes	14	15

**Table 2:** Infant Male Circumcision Belief and Opinion Statements.

**Belief and Opinion Statement Term**	**Belief and Opinion Statement Description**
SSA Belief	The research conducted in SSA on MC is relevant to the US population.
Medical Benefits Belief	The medical benefits associated with IMC are sufficient to recommend to expecting parent(s) and/or guardian(s).
Cultural Belief	The decision to circumcise a male newborn should be based on cultural, religious, and personal reasons and not on health benefits and risks.
Institutional Practice Agreement	Do you agree with studied institution’s new IMC practice? (This new institutional practice established an outpatient circumcision clinic in the pediatrics department. As a result, the obstetrics department no longer performs circumcision procedures in the hospital post-delivery.)
AAP Belief	Do you agree with AAP’s statement towards IMC? (At the time of the survey, the AAP statement (published in 1999 and reaffirmed in 2005) was considered more neutral on IMC than the current statement (published in 2012).

Univariate and multivariable regression analyses were conducted to examine the HCPs’ characteristics that are associated with knowledge, as measured by the summary score (linear regression) and the HIV item (logistic regression). Initially, we conducted univariate regressions with each predictor variable, and included those predictors with associations with *P* ≤ 0.10 in the multivariable regressions. All multivariable models were controlled for gender.

## Results

One hundred ninety-one HCPs were identified as potential participants for this study, of which 29 were not eligible to participate. The ineligible individuals did not currently practice at the studied academic health center (n = 14, 48%), were first year residents (n = 8, 28%), and/or did not treat newborns/infants or pregnant patients (n = 7, 24%).

Ninety-two participants enrolled in the study and completed the survey, generating a 57% response rate; 40% from obstetrics (n = 37), 31% from pediatrics (n = 28), and 29% from family medicine (n = 27). The population identified as 78% female (n = 72), 83% as a faculty physician/resident/fellow (n = 76), 52% Caucasian (n = 48), 50% as Christian (n = 46), 58% as married (n = 53), 44% completed medical training between 1990 and 2010 (n = 41), and 41% between 30 and 39 years of age (n = 38). For a complete description of the sample, see Table 1.

Responders and non-responders did not differ by medical specialty or position (chi-square = 1.21, *P* = 0.55 and chi-square = 3.56, *P* = .31, respectively).

### Overall MC Knowledge

Participants’ knowledge varied widely; no HCPs answered all questions correctly. The mean number of correct items was 10.52, the median equaled 11, the range was 3 to 15, and the standard deviation was 2.11. Providers were likely to answer the following items correctly: rate of severe adverse events associated with IMC (90% correct); does not protect against hypospadias (89%); protects against phimosis (76%); UTIs (69%); and penile cancer (63%). Participants were less likely to be correct for the following items: protection against cervical cancer in female partners (44%); does not prevent against Peyronie’s disease (40%); reduction in HIV acquisition (23%); protects against genital ulcer disease (19%); and protects against bacterial vaginosis in female partners (14%).

The HCPs demonstrated a good understanding of which cultural and religious groups traditionally do and do not circumcise (Jewish, 95%; Christian, 85%; Hindus, 95%; Buddhists, 95%; Hispanics, 87%); except only 45% indicated correctly that Muslims traditionally circumcise. When asked whether Medicaid reimburses for IMC in all states, only 28% of the HCPs responded ‘no’, which is the correct response (Table 3 for complete statistics).

**Table 3:** Total Participants’ Overall IMC Knowledge.

**Knowledge Item (Correct Response)**	**Correct**		**Incorrect**		**Unsure**		**Missing**	
	n	%	n	%	n	%	n	%
MC Offer Protection								
Hypospadias (No)	82	89	4	4	3	3	3	3
Phimosis (Yes)	70	76	10	11	11	12	1	1
Urinary Tract Infections(Yes)	63	69	21	23	5	5	3	3
Penile Cancer (Yes)	58	63	23	25	8	9	3	3
Cervical Cancer (Yes)	40	44	34	37	17	18	1	1
Peyronie’s Disease (No)	37	40	12	13	40	44	3	3
Genital Ulcer Disease (Yes)	17	19	39	42	33	36	3	3
Bacterial Vaginosis (Yes)	13	14	51	55	26	28	2	2
Rates								
IMC severe adverse events rates (0%–4%)	83	90	9	10	0	0	0	0
MC % reduction in HIV	21	23	69	75	0	0	2	2
acquisition (60%)								
Religious/Ethnic groups circumcise								
Jewish (Yes)	87	95	0	0	5	5	0	0
Hindu (No)	87	95	0	0	5	5	0	0
Buddhist (No)	87	95	0	0	5	5	0	0
Christian (No)	78	85	9	10	5	5	0	0
Muslim (Yes)	41	45	46	50	5	5	0	0
Hispanics IMC rates higher than whites (No)	80	87	5	5	7	8	0	0
Policy Issues								
Medicaid fully reimburses in all	26	28	26	28	40	44	0	0
50 states (No)								

### Overall Knowledge Summary Score Analysis

The following variables were associated (*P*≤.10) with greater knowledge as determined by the overall knowledge summary score in univariate linear regression analyses: participant’s medical specialty, religious affiliation, refused to perform IMC at one point in one’s career, SSA belief, and AAP belief (see Table 4).

**Table 4:** Factors Associated with Knowledge Score: Univariate Linear Regression Analysis.

**Independent Explanatory Variables**	**Parameter Estimate**	**95% CI**	***P***
Medical Specialty			
Pediatrics	0.94	0.01, 1.88	0.05
Family Medicine and Obstetrics	Ref		
Religion			
Islam/Judaism	0.23	-0.69, 1.16	0.62
Other Religious Affiliation	1.27	0.04, 2.51	0.04
Christian	Ref		
Ever Refused IMC			
Yes	0.95	0.03, 1.87	0.04
No	Ref		
SSA Belief			
Strongly Agree/Agree	0.78	-0.08, 1.65	0.07
Strongly Disagree/Disagree/Neither Disagree nor Agree	Ref		
AAP Belief			
Strongly Agree/Agree	-1.32	-2.59, 0.04	0.04
Strongly Disagree/Disagree/Neither Disagree nor Agree	Ref		
Gender			
Females	0.36	-0.71, 1.42	0.51
Males	Ref		

All of these independent explanatory variables were entered into the final model along with gender to control for any confounding issues. The final model accounted for significant variance in the summary scores (R^2^ = 0.18, adjusted R^2^ = 0.11, *P* = .02). Pediatricians scored significantly higher on the knowledge score compared to obstetrics and family medicine providers (β = 0.96, *P* = .04).Those who refused to perform IMC at one point in their careers scored 0.92 points higher on the knowledge score compared with those who had never refused (*P* = .05). All other variables we investigated were not significantly associated with the knowledge score in the final model (Table 5).

**Table 5:** Factors Associated with Knowledge Score Adjusted for Confounders.

**Multivariable Linear Regression Analysis**	** **	** **	** **
Independent Explanatory Variables	Parameter Estimate	95% CI	*P*
Medical Specialty			
Pediatrics	0.96	0.05, 1.88	0.04
Family Medicine and Obstetrics	Ref		
Religion			
Islam/Judaism	0.35	-0.62, 1.32	0.48
Other Religious Affiliation	0.84	-0.48, 2.15	0.21
Christian	Ref		
Ever Refused IMC			
Yes	0.92	0.01, 1.84	0.05
No	Ref		
SSA Belief			
Strongly Agree/Agree	0.62	-0.23, 1.47	0.15
Strongly Disagree/Disagree/Neither Disagree nor Agree	Ref		
AAP Belief			
Strongly Agree/Agree	-0.85	-2.13, 0.45	0.20
Strongly Disagree/Disagree/Neither Disagree nor Agree	Ref		
Gender			
Females	0.14	-0.91, 1.18	0.80
Males	Ref		
			
R^2^	0.18		
Adjusted R^2^	0.11		

### HIV Knowledge Score Analysis

Given that only 23% of the participants knew that MC reduces the risk of HIV by 60% and the recent attention in the medical literature, we examined this knowledge item in detail. The univariate analysis indicated that participant’s medical specialty, religious affiliation, age, decade training was completed, refused to perform IMC at one point in one’s career, and institutional practice agreement were associated with knowledge of HIV (Table 6). Decade medical training complete and age are highly correlated with one another; therefore, decade medical training complete was the only variable entered into the model to avoid multicollinearity issues.

**Table 6:** HIV Knowledge Score: Univariate Logistic Regression Analysis.

**Borderline Significant Independent Explanatory Variables**	**Unadjusted Odds Ratio**	**95% CI**	***P***
Medical Specialty			
Obstetrics	5.07	1.29–19.95	0.02
Family Medicine	1.45	0.29, 7.19	0.65
Pediatrics	Ref		
Religion			
Islam/Judaism	1.39	0.45, 4.31	0.57
Other Religious Affiliation	4.07	1.07, 15.40	0.04
Christian	Ref		
Decade Training Complete			
2000 and after	0.34	0.13, 0.94	0.03
Before 2000	Ref		
Ever Refused IMC			
Yes	3.06	1.11, 8.40	0.03
No	Ref		
Institutional Practice Agreement Item			
Strongly Agree/Agree	3.39	1.23, 9.32	0.02
Strongly Disagree/Disagree/Neither Disagree nor Agree	Ref		
Sex			
Females	0.86	0.27, 2.72	0.79
Males	Ref		

In multivariable regression, only participant’s medical specialty and institutional practice agreement were significantly associated with knowledge of HIV (*P*≤0.05). The participants in the obstetrics medical specialty were 5.28 (95% CI = .99–28.21) times more likely to answer the HIV question correctly than the pediatric providers. Those who strongly agreed or agreed with the institutional practice agreement item were less likely to answer the HIV question correctly (OR = .28, 95% CI: 0.08–1.01). (see Table 7).

**Table 7:** Factors Associated with HIV Knowledge Score Adjusted for Confounders: Multivariable Logistic Regression Analysis.

Chi-Square	23.07		
DF	8		
*P*	0.003		
Goodness of Fit	0.82		
			
Explanatory Independent Variables	Adjusted Odds Ratio	95% CI	*P*
Medical Specialty			
Obstetrics	5.28	0.99, 28.21	0.05
Family Medicine	1.85	0.26, 13.01	0.54
Pediatrics	Ref		
Religion			
Islam/Judaism	1.88	0.46, 7.45	0.38
Other Religious Affiliation	4.90	0.94, 25.51	0.06
Christian	Ref		
Decade Training Complete			
2000 and after	0.36	0.11, 1.21	0.10
Before 2000	Ref		
Ever Refused IMC			
Yes	2.26	0.68, 7.51	0.18
No	Ref		
Institutional Practice Agreement Item			
Strongly Agree/Agree	0.28	0.08, 1.01	0.05
Strongly Disagree/Disagree/Neither Disagree nor Agree	Ref		
Gender			
Females	0.78	0.15, 3.94	0.11
Males	Ref		

## Discussion

The results of this study demonstrate that significant gaps exist in HCPs’ knowledge about the risks and benefits of IMC, especially regarding results of recent research showing circumcision to have a protective effect against acquisition of HIV and certain STIs. Further, we found significant variation between HCPs, with pediatricians exhibiting greater overall knowledge of the evidence compared to obstetricians and family practitioners. Obstetricians, however, were more likely to answer the HIV knowledge item correctly compared to the other medical specialties.

To our knowledge, this is the first study of HCPs’ IMC knowledge focusing on an academic health center and one of very few conducted in the United States. In 2012, Carbery et al. reported that 22% of physicians said that they did not understand IMC’s risks and benefits well enough to counsel parents.[25] Our findings that overall knowledge of IMC is variable, combined with those of Carbery et al., are important because the CDC and the AAP recommend that HCPs deliver accurate and unbiased information to parents, guardians, and patients regarding IMC’s risks and benefits.

Participants were relatively knowledgeable about some aspects of IMC, including its protective effects against phimosis, UTIs, and penile cancer, all of which were highlighted in the AAP statement published in 1999 and reaffirmed in 2005.[41, 42] HCPs were less knowledgeable about the more recent research results, including HIV, cervical cancer, bacterial vaginosis, and genital ulcer disease. This indicates that further education is needed to enable practitioners to more effectively communicate the scientific evidence regarding IMC and MC; thereby fostering dialogue and informed decision-making with the parents and guardians.

HCP’s medical specialty surfaced as an explanatory factor for overall knowledge and HIV knowledge item as mentioned previously. Pediatricians scored higher for overall IMC knowledge compared to those in obstetrics and family medicine; whereas, obstetricians were more knowledgeable about circumcision’s protective effects against HIV acquisition. These differences between medical specialties at this academic health center may be related to the center’s practice of allocating IMC procedures to pediatricians, who perform the procedure at an outpatient facility once a week. Consequently, the pediatricians may be motivated to be up-to-date with the most current research and guidelines that are associated with newborns and infants. Whereas obstetricians treat predominantly adult females, they may be more focused on remaining current regarding results of research related to HIV acquisition.

HCPs that had ever refused to circumcise a newborn or infant boy were significantly more knowledgeable than those who had never refused. The reasons that HCPs refused to circumcise were not related to cultural or religious beliefs, but stemmed from medical indications that create a risk with the procedure (e.g., hypospadias, penile abnormalities, small penis size, and concern for sepsis). In order to make an informed unbiased decision about IMC and to counsel parents accordingly, the HCP must be familiar with the associated risks as well as the medical and health benefits of the procedure.

Educational intervention is warranted to increase HCP’s level of knowledge with the support of institutional organizations and governing medical bodies as well as policy makers. Past research indicates that in order to systemically promote the translation of knowledge, all decision makers including patients, HCPs, institutional administrators, and policy makers must have access to current evidence-based research.[30]

The educational intervention should be specific to the clinic or institution depending on their IMC protocols. To increase HCPs’ knowledge and promote informed decision-making, a multi-prong approach is necessary. This approach may include the following: (1) the creation of a module about IMC for HCPs through a continuing medical education course and/or implemented within HCPs’ training curriculum (2) distributing AAP’s statement about male circumcision to HCPs and discussing this at staff meetings and/or (3) creating fact sheets and pamphlets for HCPs and parent(s)/guardian(s) to increase their level of knowledge and facilitate discussion.

In the case of IMC, institutional support is necessary to engender an environment which allows HCPs to communicate accurate and unbiased information to promote informed decision-making processes.[43, 44] The AAP recommends that professional organizations including the ACOG, the American Academy of Family Practitioners, the American Urological Association, the American Society of Anesthesiologists, and the American College of Nurse Midwives should work together with the AAP to develop educational materials and standards of trainee proficiency, and ensure inclusion of IMC procedural techniques in postgraduate training programs.[28] This collaboration will also help in fostering the dialogue at the institutional level as well as targeting providers at the individual level.

Our study has several limitations. The sample is drawn from a single institution in one geographical location with very specific processes for performing IMC, and therefore, limits the generalizability outside of the studied population. Secondly, the response rate was 57% for the online survey, and this might not represent the full range of HCPs. While nonresponse bias could be present, we found no significant differences between non-responders and responders for position and medical specialty. The confidence intervals for some results were wide; a larger sample size would result in more precise estimates. Further, the results indicate that there may be other factors that we did not measure that explain additional variance in HCPs’ IMC knowledge. The knowledge score was constructed incorporating current evidence-based literature in collaboration with MC experts. The authors acknowledge that this is not the only way to measure IMC knowledge, and as a result, we have clearly outlined how we measured this construct in detail within the manuscript.

## Conclusions

The results of this study indicate that there is considerable variation in level of IMC knowledge among providers. We recommend an IMC system-based educational intervention for HCPs involved in the IMC decision process focused on increasing knowledge and working in collaboration with health centers, hospitals, healthcare accreditation organizations, and medical bodies. Dissemination of the AAP’s IMC statement coupled with educational materials and other interventions will promote systemic knowledge translation and facilitate informed, evidence-based decision-making processes for parents and guardians.
